# Liraglutide suppresses non-esterified free fatty acids and soluble vascular cell adhesion molecule-1 compared with metformin in patients with recent-onset type 2 diabetes

**DOI:** 10.1186/s12933-018-0701-4

**Published:** 2018-04-10

**Authors:** Xiao-min Chen, Wen-qiang Zhang, Yuan Tian, Li-fen Wang, Chan-chan Chen, Chuan-mei Qiu

**Affiliations:** 1 Department of Endocrinology and Metabolism, Zhongshan Hospital Xiamen University, 201-209 Hubin South Road, Xiamen, 361004 People’s Republic of China; 2 Guangzhou Medicine University Second Affiliated Hospital, 250-296 Changgang East Road, Guangzhou, 510260 People’s Republic of China

**Keywords:** Liraglutide, Metformin, NEFA, sVCAM-1, Type 2 diabetes

## Abstract

**Background:**

It has been suggested that liraglutide could have an impact on glucose and lipid metabolism disorder and adhesion molecule activation, which may play important roles in the vascular damage of diabetes. In this study, we examined the effects of liraglutide versus metformin on non-esterified free fatty acids, beta-cell insulin secretion, and adhesion molecule levels in patients with recent-onset type 2 diabetes mellitus.

**Methods:**

In this study, 60 patients newly diagnosed with type 2 diabetes mellitus (mean age 33.97 ± 5.67 years) were randomly assigned to receive once-daily subcutaneous liraglutide or oral metformin. Before the study and after the 8-week treatment period, a 75 g oral glucose tolerance test was performed. Plasma glucose, lipids and lipoprotein, plasma insulin, glycaemic and insulin responses, non-esterified free fatty acids (NEFA), and soluble vascular cell adhesion molecule-1 (sVCAM-1) levels were evaluated.

**Results:**

After 8 weeks, 120 min of NEFA (155 ± 125 vs 99 ± 73 µmol/L, *P *= 0.026) and the levels of sVCAM-1 (465 ± 136 vs 382 ± 131 ng/ml, *P *= 0.013) significantly decreased, while the early phase insulin secretion index (24.94 [7.78, 38.89] vs. 31.13 [17.67, 59.09], *P *= 0.031), fasting plasma insulin (104 [51, 123] vs 113 [54, 171] mIU/L, *P *= 0.015), 60 min plasma insulin (326 [165, 441] vs 471 [334, 717] mIU/L, *P *= 0.005), 120 min plasma insulin (401 [193, 560] vs 500 [367, 960] mIU/L, *P *= 0.047), and insulin area under the curve (AUCins) (648 [321, 742] vs 738 [451, 1118] mIU/L, *P *= 0.005) remarkably increased for patients in the liraglutide treatment group. The levels of sVCAM-1 dramatically decreased after 8 weeks of liraglutide treatment (503 ± 182 vs 382 ± 131 ng/ml, *P *= 0.046) compared to that of the metformin treatment group. At the same time, the differences before and after liraglutide treatment in 120 min of NEFA (− 32 [− 96, − 5] vs 5 [− 35, 38] µmol/L, *P *= 0.033) and AUCins (738 [451, 1118] vs 594 [357, 1216] mIU/L, *P *= 0.014) were remarkably enhanced compared to that of the metformin therapy. Nevertheless, there were no significant differences in fasting NEFA after liraglutide or metformin treatment. The reduction of 120 min NEFA (ΔNEFA) was positively correlated with the decrease of sVCAM-1 (ΔsVCAM-1) after 8 weeks of liraglutide treatment (*r *= 0.523, *P *= 0.003).

**Conclusions:**

Our results demonstrate that liraglutide administration is more effective than metformin in reducing 120 min NEFA and suppressing sVCAM-1 levels for recent-onset type 2 diabetes mellitus. We suggest that this outcome may be because liraglutide is associated with potentiating insulin secretion capacity, inhibiting vascular inflammatory cytokines, and antagonizing atherosclerosis.

**Electronic supplementary material:**

The online version of this article (10.1186/s12933-018-0701-4) contains supplementary material, which is available to authorized users.

## Introduction

It has been well documented that atherosclerosis is the major cause of death in type 2 diabetic patients [1, 2]. Dyslipidaemia plays an important role in the pathogenesis of atherosclerosis and even to insulin resistance [3, 4]. Dyslipidaemia is associated with increased lipolysis and the release of higher amounts non-esterified fatty acids (NEFAs) into the bloodstream [5]. Hyperglycaemia promotes lipolysis and leads to chronic exposure to NEFA [6]. Several studies have shown that the elevated serum level of NEFA contributes to vascular damage in diabetes [7, 8].

Plasma NEFAs promote a systemic insulin resistance state and are correlated with inflammation in atheromatous plaques [5, 7]. The levels of NEFAs seem to be modified by dietary or therapeutic intervention. Glucagon-like peptide-1 (GLP-1) has recently received attention as a novel drug in the antidiabetic field [9]. The long-acting GLP-1 analogue liraglutide (LRG) exhibits pleiotropic effects on glucolipid metabolism, β-cell insulin secretion and anti-atherosclerosis [10–15]. Nevertheless, the precise mechanism by which liraglutide modulates lipids metabolism remains unclear.

Therefore, the aim of this study is to evaluate whether liraglutide could be more effective at suppressing lipolysis, ameliorating glucose and lipid metabolism, enhancing beta-cell functions, and inhibiting vascular inflammatory markers compared to metformin in recent-onset type 2 diabetes mellitus.

## Subjects and methods

### Ethical approval

Ethical approval was granted by the Institutional Review Board of Xiamen University Affiliated Zhongshan Hospital according to Helsinki Declaration II. Written informed consent was signed by and obtained from each participant.

### Participants

Participants were recruited between October 2015 and December 2016 at Zhongshan Hospital Xiamen University in P. R. China. The inclusion criteria were set to select participants: the patients had an initial diagnosis of type 2 diabetes mellitus who met the World Health Organization criteria for type 2 diabetes and were aged 18–40 years, with a body mass index (BMI) of 25–35 kg/m^2^, HbA1c of 6.5–9%, and fasting serum triglycerides of 0.5–5 mmol/L. Main exclusion criteria included the following: type 1 diabetes, recent acute complications including diabetic ketoacidosis and hyperglycaemic hyperosmolar state, impaired liver function, impaired renal function (creatinine clearance < 60 ml/min) [16], pregnancy and breast bleeding, and other factors that affect glucose and lipid changes.

### Study design and procedures

This study is a randomized, parallel, active comparator trial in 60 participants. The treatment order of liraglutide or metformin was determined through random assignment (1:1). All individuals who received liraglutide treatment started injection doses at 0.6 mg/day. After 1 week of injection, the dose was increased to 1.2 mg/day. Participants in another group who received metformin received an oral dose in the range of 1–2 g/day. After an overnight fast, a 75 g oral glucose tolerance test (OGTT) was conducted for each participant. Blood samples were drawn before and 30, 60, 120 min after OGTT, respectively. Participants in the liraglutide treatment group were instructed to administer the Flexpen device. Participants were also informed of medication precautions before metformin treatment. All participants received lifestyle intervention health education from professional nurse. The total planned treatment period was 8 weeks. There were a total of 3 follow-up visits, once per month. The plasma glucose, blood pressure, body weight, waist circumference and hip circumference were measured, and adverse events were monitored by investigators. At the end of the trial, the clinical and laboratory indices were assessed, as previously described.

### Clinical measurement and laboratory test

Weight, waist circumference (WC), and hip circumference were measured by professional nurses. Participants removed clothing and shoes prior to measurements. WC was measured midway between the lowest rib and the top of the iliac crest. Hip circumference was measured around the peak of the buttocks. Body mass index (BMI) was calculated as the body weight (BW) in kilograms divided by the square of the patient’s height in metres. Measurements of systolic blood pressure and diastolic blood pressure (SBP and DBP) were performed 2 times with a mercury manometer on the right arm after 5 min in the sitting position. The mean values of measurements were recorded.

Participants were instructed to refrain from excessive physical exercise and to eat normally 3 days before the test, with no less than 200 grams of carbohydrates daily. After fasting overnight (8–10 h), a 2-h OGTT test was performed on each subject. Fasting plasma glucose (FPG), 30 min PG, 60 min PG, and 120 min PG were measured by the hexokinase method. Subjects’ renal and liver functions, plasma lipids and lipoprotein concentrations including triglycerides (TG), total cholesterol (TC), low-density lipoprotein cholesterol (LDL-C), and high-density lipoprotein cholesterol (HDL-C) were assayed using standard methods (Roche cobas8000 automatic biochemical analyser). HbA1c was analysed by HPLC (Bio-Rad, Inc., Hercules, CA, USA). Plasma insulin levels were measured using the electrochemiluminescence immunoassay (ECLI). NEFA levels were measured in the Xiamen Diabetes Hospital using Abbott c16000 automatic biochemical analyser. Soluble vascular cell adhesion molecule-1 (sVCAM-1) and soluble intercellular adhesion molecule-1 (sICAM-1) were measured in the plasma of patients using quantikine ELISA kits (R&D systems, Inc., Minneapolis, USA). Plasminogen activator inhibitor 1 (PAI-1) concentrations were assessed using ELISA assays (Multi sciences, Inc., Hangzhou, PRC). The intra-assay and inter-assay coefficients of variation (CV) of the ELISA kits mentioned above were all less than 10%. The early phase insulin secretion index was calculated as (ΔI30/ΔG30) = ([insulin at 30 min] − [insulin at 0 min])/([glucose at 30 min] − [glucose at 0 min]) [17]. The area under the curve (AUC) for plasma insulin during the OGTT was calculated using the trapezoidal rule [18]. Deltas (Δ) are presented as the difference before and after treatment, which were available for the variables ΔNEFA, ΔsVCAM-1, ΔAUCins, and ΔI30/ΔG30. Deltas were also considered to express the difference between 120 min values and fasting values in ΔTC and ΔLDL-C.

### Statistical analysis

SPSS version 21 (SPSS software, IBM Inc., USA) and GraphPad Prism version 5.0 (GraphPad software, Inc., La Jolla, CA, USA) were utilized for statistical analysis and the construction of graphs. The normal distribution data are presented as the mean ± standard deviation. Comparisons of basic characteristics between groups were made using an unpaired *t* test. Comparisons of baseline and post-treatment data in the same group were made using a paired *t* test. The skewed distribution variables are expressed as the median (interquartile rang), and the Mann–Whitney *U* test or Wilcoxon signed rank test was performed. The Mann–Whitney *U* test was used to determine the differences from baseline with a final measurement at 8 weeks for 120 min NEFA, insulin secretion capacity including fasting insulin, 30 min insulin, 60 min insulin, 120 min insulin, ΔI30/ΔG30, and AUCins between the liraglutide and metformin group. At the same time, the Mann–Whitney *U* test was carried out to determine the differences between 120 min values and fasting values on TC and LDL-C in the metformin group. Spearman rank correlations were performed to determine the relationship of ΔNEFA with ΔsVCAM-1, ΔAUCins and ΔI30/ΔG30 after 8 weeks of treatment. The Chi squared (*χ*^*2*^) test was used for categorical variables. Two-tailed significance was set at *P *< 0.05.

## Results

### Comparisons of clinical and laboratory characteristics of the study participants

The patient characteristics grouped by either liraglutide or metformin treatment are presented in Table 1. In the baseline analysis, the differences in participants’ age, BW, BMI, SBP, DBP, WC and hip circumference between the two groups were not statistically significant. HbA1c, FPG, 60 min PG, 120 min PG, NEFA, lipids and lipoprotein concentrations (including TC, TG, LDL-C, HDL-C), insulin secretion capacity, sVCAM-1, sICAM-1, and PAI-1 were similar between the two groups, but 30 min PG was higher at baseline in the liraglutide treatment group.

**Table 1:** Baseline characteristics of the study participants

Variable	Liraglutide (n = 30)	Metformin (n = 30)	*P*-value
Age (years)	32.67 ± 5.46	35.27 ± 5.85	0.219
Male/female (n)	20/10	20/10	1.000
BW (kg)	81 ± 17	72 ± 11	0.104
BMI (kg/m^2^)	28.63 ± 3.86	26.16 ± 3.1	0.063
Waist circumference (cm)	92.20 ± 11.99	88.27 ± 8.22	0.304
Hip circumference (cm)	99.87 ± 6.02	96.33 ± 5.77	0.112
SBP (mmHg)	122 ± 16	120 ± 11	0.643
DBP (mmHg)	79 ± 12	81 ± 7	0.719
Cholesterol (mmol/L)			
Fasting value	4.79 ± 0.87	5.02 ± 0.79	0.444
120 min value	4.45 ± 1.02	4.55 ± 0.81	0.777
Triglycerides (mmol/L)			
Fasting value	1.82 ± 0.74	1.99 ± 0.74	0.539
120 min value	2.07 ± 1.23	1.97 ± 0.68	0.802
LDL-C (mmol/L)			
Fasting value	3.61 ± 0.82	3.78 ± 0.68	0.535
120 min value	3.16 ± 0.70	3.28 ± 0.70	0.639
HDL-C (mmol/L)			
Fasting value	1.12 ± 0.29	1.20 ± 0.26	0.448
120 min value	1.04 ± 0.35	1.07 ± 0.23	0.793
HbA_1_c (%)	8.49 ± 1.81	7.77 ± 1.44	0.238
FPG (mmol/L)	9.40 ± 2.32	8.45 ± 1.57	0.198
30 min PG (mmol/L)	15.43 ± 2.96	13.03 ± 2.72	0.028
60 min PG (mmol/L)	18.19 ± 3.60	16.43 ± 4.13	0.224
120 min PG (mmol/L)	17.68 ± 4.38	14.47 ± 5.05	0.074
Fasting insulin (mIU/L)	104 (51, 123)	76 (60, 150)	0.724
30 min insulin (mIU/L)	271 (136, 298)	211 (118, 444)	0.917
60 min insulin (mIU/L)	326 (165, 441)	368 (242, 731)	0.152
120 min insulin (mIU/L)	401 (193, 560)	475 (286, 1060)	0.178
AUCins (mIU/L)	648 (321, 742)	615 (381, 1167)	0.520
ΔI30/ΔG30	24.94 (7.78, 38.89)	30.18 (10.4, 53.75)	0.272
NEFA (μmol/L)			
Fasting value	620 ± 468	485 ± 225	0.325
120 min value	155 ± 125	101 ± 53	0.309
sVCAM-1 (ng/ml)	465 ± 136	485 ± 122	0.669
sICAM-1 (ng/ml)	113 ± 40	168 ± 110	0.085
PAI-1 (ng/ml)	69.52 ± 45.75	51.34 ± 38.87	0.227

Data are expressed as mean ± standard deviation or median (interquartile rang)

*BW* body weight, *BMI* body mass index, *SBP* systolic blood pressure, *DBP* diatolic blood pressure, *LDL-C* low-density lipoprotein cholesterol, *HDL-C* high-density lipoprotein cholesterol, *HbA*_*1*_*c* glycated haemoglobin, *FPG* fasting plasma glucose, *AUCins* insulin area under the curve, *NEFA* non-esterified fatty acids, *sVCAM-1* soluble vascular cell adhesion molecule-1, *sICAM-1* soluble intercellular adhesion molecule-1, *PAI-1* plasminogen activator inhibitors-1

After 8-week metformin treatment, fasting total cholesterol (TC) and low density lipoprotein cholesterin (LDL-C) were remarkably lower compared to baseline (5.02 ± 0.79 vs 4.45 ± 0.28 mmol/L, *P *= 0.006), (3.78 ± 0.68 vs 3.18 ± 0.88 mmol/L, *P *= 0.012). At the same time, metformin therapy also reduced 120 min TC (4.55 ± 0.81 vs 4.10 ± 1.21 mmol/L, *P *= 0.036) and 120 min LDL-C (3.28 ± 0.7 vs 2.76 ± 0.91 mmol/L, *P *= 0.008) after OGTT. However, in the present study, there were no significant differences between 120 min values and fasting values in ΔTC and ΔLDL-C with respect to metformin treatment. Furthermore, no significant differences were found in plasma lipids and lipoprotein after liraglutide treatment compared with the basal values (shown in Additional file 1: Table S1).

We found a significant reduction in waist circumference (92 ± 12 vs 88 ± 11 cm, *P *= 0.001) and body weight (81 ± 17 vs 78 ± 16 kg, *P *= 0.001), as well as BMI (28.63 ± 3.86 vs 27.67 ± 3.62 kg/m^2^, *P *= 0.001), with liraglutide treatment. Metformin treatment also showed a slight downtrend in BW and BMI, although the difference was not significant (shown in Additional file 1: Table S2).

### Liraglutide treatment elevated insulin secretion capacity and decreased plasma glucose

Early phase insulin secretion index (ΔI30/ΔG30) (24.94 [7.78, 38.89] vs 31.13 [17.67, 59.09], P = 0.031), fast plasma insulin (104 [51, 123] vs 113 [54, 171] mIU/L, *P *= 0.015), 60 min plasma insulin (326 [165, 441] vs 471 [334, 717] mIU/L, *P *= 0.005), 120 min plasma insulin (401 [193, 560] vs 500 [367, 960] mIU/L, *P *= 0.047), and insulin area under the curve (AUCins) (648 [321, 742] vs 738 [451, 1118] mIU/L, P = 0.005) significantly increased with liraglutide treatment. The differences in AUCins (ΔAUCins) before and after liraglutide treatment (738 [451, 1118] vs 594 [357, 1216] mIU/L, P = 0.014) were remarkably enhanced compared to that in metformin therapy, but differences in ΔI30/ΔG30 before and after liraglutide treatment (6.01 [1.79, 28.46] vs − 2.62 [− 14.85, 8.76], P = 0.065) were not significant between the two groups (Table 2). In fact, both liraglutide (8.49 ± 1.81 vs 6.92 ± 0.81%, *P *= 0.002) and metformin treatment (7.77 ± 1.44 vs 6.43 ± 0.71%, *P *= 0.001) could reduce HbA1c values. FPG, 30 min PG, 60 min PG and 120 min PG all decreased after liraglutide treatment, but only FPG was significantly reduced after metformin treatment (shown in Additional file 1: Table S2).

**Table 2:** Comparisons of sVCAM-1, 120 min NEFA, AUCins and ΔI30/ΔG30 levels after 8-week treatment between liraglutide and metformin group

Variable	Liraglutide	Metformin	Difference	P-value
sVCAM-1 (ng/ml)	382 ± 131	503 ± 182		0.046
120 min NEFA (μmol/L)^a^	− 32 (− 96, − 5)	5 (− 35, 38)	− 20.5 (− 46.5, 31.5)	0.033
AUCins (mIU/L)^a^	738 (451, 1118)	594 (357, 1216)	39 (− 33, 227)	0.014
ΔI30/ΔG30^a^	6.01 (1.79, 28.46)	− 2.62 (− 14.85,8.76)	4.56 (− 4.82, 12.99)	0.065

Data are expressed as mean ± standard deviation or median (interquartile rang)

*sVCAM-1* soluble vascular cell adhesion molecule-1, *NEFA* non-esterified fatty acids, *AUCins* insulin area under the curve

ΔI30/ΔG30 = [(insulin at 30 min) − (insulin at 0 min)]/[(glucose at 30 min) − (glucose at 0 min)]

^a^Difference between pre-treatment and post-treatment

### Liraglutide treatment inhibited 120 min non-esterified free fatty acids

**Fig. 1:**
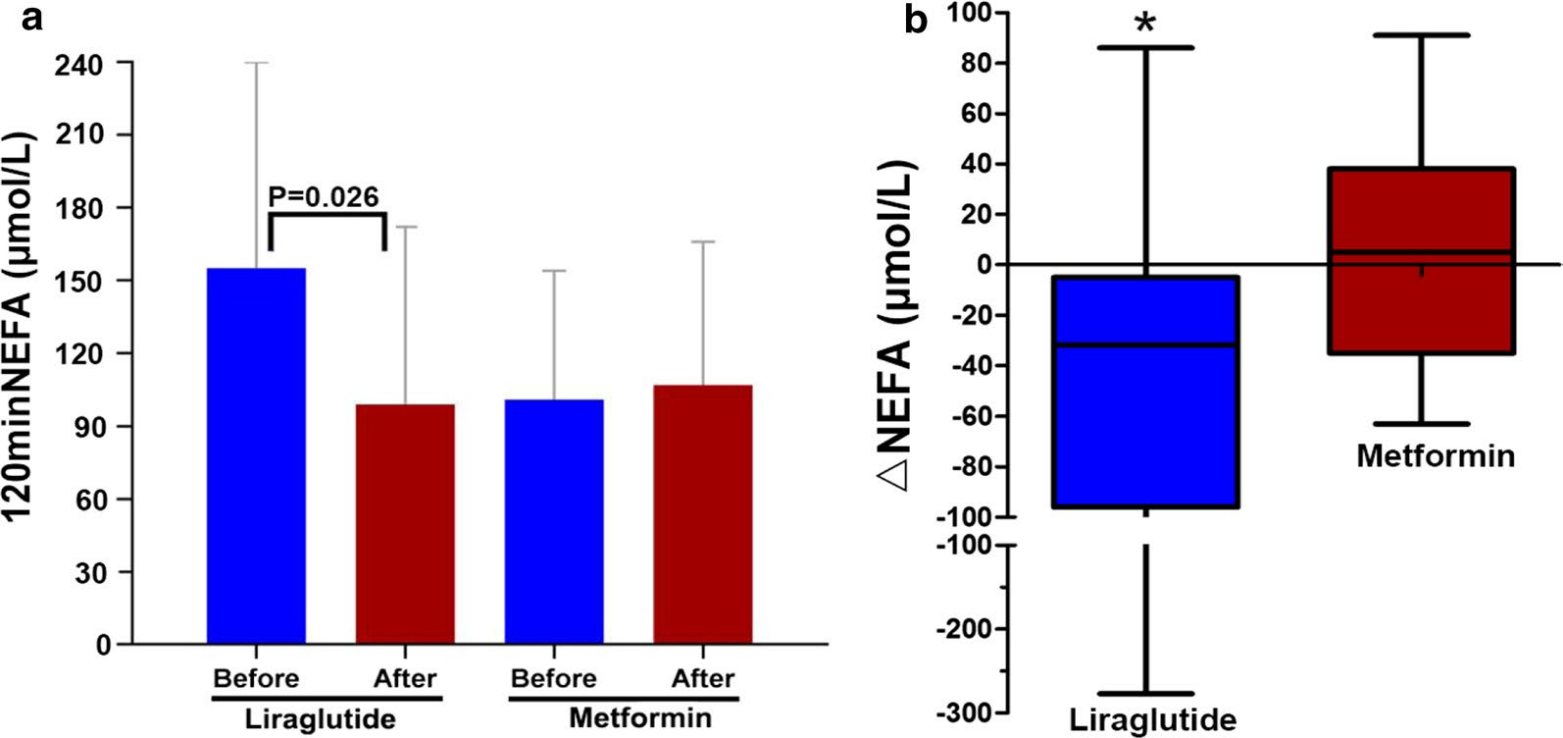
**a** 120 min non-esterified fatty acid (NEFA) before and after 8-week treatment with liraglutide and metformin. **b** The difference of NEFA (∆ NEFA) between baseline and after 8-week treatment with liraglutide and metformin *P <0.05 vs the metformin group

After 8 weeks, there was a progressive decrease in the levels of 120 min NEFA (155 ± 125 vs 99 ± 73 µmol/L, *P *= 0.026) after liraglutide treatment. Compared with metformin therapy, the reduction in 120 min NEFA (ΔNEFA) remarkably increased after 8-week liraglutide treatment [− 32 (− 96, − 5) vs 5 (− 35, 38) µmol/L, P = 0.033] (Table 2). The changes in 120 min NEFA showed a slight increase after metformin therapy (Fig. 1). There were no significant changes in fasting non-esterified free fatty acids after liraglutide and metformin treatment.

### Liraglutide treatment inhibited vascular inflammatory marker levels

**Fig. 2:**
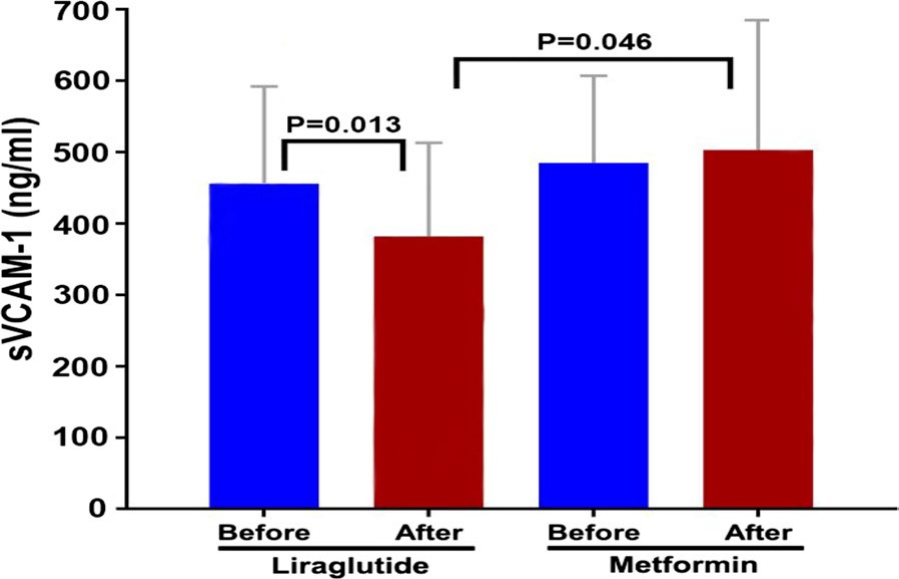
Changes of soluble vascular cell adhesion molecule-1 (sVCAM-1) before and after 8-week treatment with liraglutide and metformin

We found a significant reduction in sVCAM-1 (465 ± 136 vs 382 ± 131 ng/ml, *P *= 0.013) after liraglutide treatment compared with baseline. Compared to metformin therapy, the levels of sVCAM-1 were remarkably suppressed after 8-week liraglutide treatment (382 ± 131 vs 503 ± 182 ng/ml, *P *= 0.046) (Fig. 2, Table 2). However, there were no significant differences in sICAM-1 and PAI-1 levels before and after liraglutide and metformin therapy (shown in Additional file 1: Table S3). In all subjects combined, the reduction of 120 min NEFA (ΔNEFA) was positively correlated with the decrease of sVCAM-1 (ΔsVCAM-1) after 8-week liraglutide treatment (*r *= 0.523, *P *= 0.003), which was not significantly negatively correlated with the increase of AUCins (ΔAUCins) (*r *= − 0.286, *P *= 0.125) and ΔI30/ΔG30 (*r *= − 0.150, *P *= 0.429) (shown in Additional file 1: Table S4).

## Discussion

Our results indicate that prolonged liraglutide treatment for 8 weeks effectively inhibited 120 min non-esterified free fatty acids (NEFAs). We utilized a 75 g oral glucose tolerance test (OGTT) to evaluate the changes of NEFA, which should avoid the effects of a high-fat diet on postprandial lipid metabolism. As observed in this study, NEFA levels gradually decreased after glucose uptake, and 120 min NEFAs are dramatically lower than fasting NEFAs. Furthermore, our data show that liraglutide exhibits greater stimulation on the insulin secretion capacity, including early phase insulin secretion, fast plasma insulin, 60 min plasma insulin, 120 min plasma insulin and insulin area under the curve (AUCins) relative to those at baseline. Previous research has shown that insulin could suppress chylomicron synthesis from human jejunal explants [19]. Additionally, circulating FFAs derived from chylomicron are reduced in the hyperinsulinaemia state, as insulin prevents lipolysis in the regulation of hepatic and intestinal lipoprotein production [20, 21].

Our data demonstrated that after the 8-week treatment period, the reduction in 120 min NEFA (ΔNEFA) was greater increased, and the increase in AUCins (ΔAUCins) was also enhanced in liraglutide treatment relative to metformin treatment. Consequently, in this study, liraglutide therapy exhibited beneficial effects in ameliorating the β-cell secretion capacity and suppressing free fatty acid production. However, the precise mechanisms by which glucagon-like peptide-1 affects lipids metabolism are less certain. It has been hypothesized that enterocyte GLP-1 receptor signalling is essential for postprandial lipoprotein synthesis and secretion, reduces intestinal lipid production and absorption, and prevents the postprandial increases in triacylglycerol, cholesterol and apo-B48 levels [22]. Moreover, GLP-1 is related to the direct modulation of fatty acid binding protein 2 (FABP2), which is required for the formation of apo-B48 containing chylomicrons [23]. In addition, even through enhanced insulin secretion, GLP-1 induced prolonged reductions in NEFA concentrations after meals, suppressed postprandial rises in ApoCIII [24]. ApoCIII is a small glycoprotein synthesized in the liver and intestine that resides predominantly on the surface of ApoB-containing lipoproteins and HDL. ApoCIII inhibits lipoprotein lipase activity and interferes with the receptor-mediated uptake of TG-rich lipoproteins, therefore delaying the clearance of TG-rich lipoproteins [25, 26]. On the other hand, a long-term increase in NEFA levels has been associated with ROS accumulation, which causes mitochondrial stress and leads to β-cell apoptosis [27, 28]. In a catch-up growth rat model by re-feeding with high-fat diet, liraglutide prevented the increase of plasma NEFA, increased insulin secretion, increased islet pancreatic duodenal homeobox-1 (Pdx-1) and B cell lymphoma-2 (Bcl-2) expression, and reduced procaspase-3 transcription and Caspase-3 p11 subunit protein expression, which suggested that liraglutide treatment could antagonize β-cell apoptosis caused by elevated NEFA [11].

Dyslipidaemia has been reported to affect vascular endothelial function by the inflammatory pathway [29]. Raised non-esterified fatty acids impair insulin’s effect on glucose uptake in skeletal muscle and could thus have detrimental effects on the vascular endothelium, which leads to premature cardiovascular disease [30]. As reported previously, the adhesion molecules usually act as biomarkers, which mediate interactions between leukocytes and the vascular endothelium, shed from cell surfaces and become soluble isoforms in the blood, namely, sVCAM-1 and sICAM-1 [31]. Our study has shown that the levels of the vascular inflammatory marker sVCAM-1 are dramatically suppressed after liraglutide treatment compared to metformin treatment. However, we did not identify appreciable differences in the levels of sICAM-1 and PAI-1 after liraglutide therapy.

Considerable evidence has demonstrated that sVCAM-1 plays an important role in the pathophysiological mechanisms of atherosclerosis [32]. During a 10-year follow-up, a longitudinal study revealed that elevated circulating sVCAM-1 concentrations in females with a history of gestational diabetes mellitus (GDM) not only represented the earliest high risk stage for developing type 2 diabetes, but also reflected the early stage of the pathway for the manifestation of future cardiometabolic disorders [33]. It is widely known that atherosclerosis leads to macroangiopathy and is responsible for most deaths in patients with diabetes [34]. Liraglutide was shown to significantly reduce the risk of major cardiovascular events in the large prospective LEADER trial [12, 35–37]. However, the precise mechanisms behind the antiatherogenic effect of liraglutide are not entirely clear, although several hypotheses have been proposed. First, it has been shown that in cultured human aortic endothelial cells (HAECs), liraglutide reduces the protein expression of VCAM-1 in response to TNFα and LPS stimulation and increases intracellular calcium and cAMP levels, leading to the phosphorylation of AMPK, which is an evolutionarily conserved fuel and stress-sensing enzyme that can be activated by calmodulin dependent protein kinase kinase-b (CAMKKb), and then downregulates monocyte adhesion, which presumably inhibits atherogenesis [38, 39]. Second, in a cellular model of human umbilical vein endothelial cells (HUVECs) obtained from umbilical cords of women affected by GDM, liraglutide exposure significantly mitigated TNFα induced endothelial monocyte adhesion as well as VCAM-1 and ICAM-1 expression, reduced the phosphorylation of the MAPK42/44 signalling pathway, inhibited NF-kB p65 nuclear translocation, and decreased peroxynitrite levels and endothelial microvesicle (EMV) release [40]. Third, after 8-week metformin treatment, TC and LDL-C were remarkably lower compared to baseline, but no significant differences were found between 120 min values and fasting values in ΔTC and ΔLDL-C. Moreover, no significant differences were found in plasma TC and LDL-C after liraglutide treatment. We presumed that liraglutide exert antiatherogenic mechanism, which might be not related to a decrease of LDL-C levels but might be related to an enhancement of HDL anti-inflammatory capacity [41].

We carried out a Spearman rank correlation assay and the data showed that the reduction of 120 min NEFA (ΔNEFA) was positively correlated with the decrease of sVCAM-1 (ΔsVCAM-1) after 8-week liraglutide treatment. Our results suggest that liraglutide has beneficial effects on suppressing plasma NEFA as well as sVCAM-1 levels, which is likely to indicate that liraglutide could protect the endothelia by inhibiting monocyte cell adhesion and sVCAM-1 activation and reducing lipocyte oxidative stress and free fatty acid production, revealing an antiatherogenic effects [42, 43].

The strengths of this study include the randomized, parallel, active comparator design and the similarities between the liraglutide and metformin groups at baseline. To the best of our knowledge, this is the first study to verify that liraglutide treatment is more effective than metformin treatment for recent-onset type 2 diabetes mellitus in reducing NEFA after glucose intake and suppressing sVCAM-1 levels at the same time. However, this study has certain limitations. First, it has a non-blinded design, lacks a non-treatment control group, and has a small sample size for the study. Second, further studies are needed to reveal the relevant signalling pathways by which liraglutide might influence glucose and lipid metabolism, regulate vascular endothelial function, and antagonize atherosclerosis.

## Conclusions

In brief, this study demonstrated that liraglutide administration is more effective in diminishing 120 min NEFAs and suppressing sVCAM-1 levels than metformin in young patients with recent-onset type 2 diabetes mellitus. This outcome could be associated with potentiating insulin secretion capacity and antagonizing vascular inflammatory cytokines. Therefore, these results suggest that liraglutide may exert a protective effect on alleviating vascular damage, antagonizing atherosclerosis, and reducing the risk of cardiovascular disease in the course of type 2 diabetes.

## Additional file

**Additional file 1: Table S1.** Changes of plasma lipids and lipoprotein metabolism parameters before and after 8-week treatment with liraglutide and metfotmin. **Table S2.** Comparisons of body weight, plasma glucose and insulin secretion capacity before and after 8-week treatment between two groups. **Table S3.** Comparisons of sVCAM-1,sICAM-1,PAI-1 levels before and after 8-week treatment between two groups. **Table S4.** Spearman rank correlations in ΔNEFA with ΔsVCAM-1, ΔAUCins and ΔI30/ΔG30 after 8-week treatment.

## Abbreviations

BMI: body mass index
WC: waist circumference
TC: total cholesterol
TG: triglycerides
HDL-C: high-density lipoprotein cholesterol
LDL-C: low-density lipoprotein cholesterol
Apo: apolipoprotein
FPG: fasting plasma glucose
HbA_1_c: glycated haemoglobin
SBP: systolic blood pressure
DBP: diastolic blood pressure
OGTT: oral glucose tolerance test
GLP-1: glucagon-like peptide-1
LRG: liraglutide
MET: metformin
NEFA: non-esterified fatty acids
sVCAM-1: soluble vascular cell adhesion molecule-1
sICAM-1: soluble intercellular adhesion molecule-1
PAI-1: plasminogen activator inhibitors-1
ELISA: enzyme-linked immunosorbent assay
HPLC: high performance liquid chromatography
ECLI: electrochemiluminescence immunoassay
CV: coefficients of variation
ROS: reactive oxygen species
GDM: gestational diabetes mellitus
HAECs: human aortic endothelial cells
AMP: activated protein kinase
AMPK: adenosine monophosphate activated protein kinase
CAMKKb: calmodulin dependent protein kinase kinase-b
TNF-α: tumor necrosis factor-α
LPS: lipopolysaccharide
HUVEC: human umbilical vein endothelial cells
MAPK: mitogen-activated protein kinase
NF-kB: nuclear factor kappa-light-chain-enhancer of activated B cells
EMV: endothelial microvesicle
