# Supplementary material for: Liraglutide suppresses non-esterified free fatty acids and soluble vascular cell adhesion molecule-1 compared with metformin in patients with recent-onset type 2 diabetes
Source: Cardiovasc Diabetol. 2018 Apr 10;17:53. doi: 10.1186/s12933-018-0701-4 (PMC5891985; doi:10.1186/s12933-018-0701-4)
Supplement: Supplementary file 1 — Additional file 1: Table S1. Changes of plasma lipids and lipoprotein metabolism parameters before and after 8-week treatment with liraglutide and metfotmin. Table S2. Comparisons of body weight, plasma glucose and insulin secretion capacity before and after 8-week treatment between two groups. Table S3. Comparisons of sVCAM-1,sICAM-1,PAI-1 levels before and after 8-week treatment between two groups. Table S4. Spearman rank correlations in ΔNEFA with ΔsVCAM-1, ΔAUCins and ΔI30/ΔG30 after 8-week treatment. [file 12933_2018_701_MOESM1_ESM.zip › Table S4.doc]

**Additional file 1: Table S4 Spearman rank correlations in ΔNEFA with ΔsVCAM-1, ΔAUCins and ΔI30/ΔG30 after 8-week treatment**

| **Variables** | **Correlation coefficients with ΔNEFA ( *r*)** | ***P*-value** |
| --- | --- | --- |
| ΔsVCAM-1(ng/ml) | 0.523 | 0.003 |
| ΔAUCins(mIU/L) | -0.286 | 0.125 |
| ΔI30/ΔG30^＊^ | -0.150 | 0.429 |

Deltas(Δ) are presented as the difference of variables before and after treatment

sVCAM-1:soluble vascular cell adhesion molecule-1; NEFA:non-esterified fatty acids; AUCins: insulin area under the curve; ΔI30/ΔG30= [(insulin at 30 min) - (insulin at 0 min)]/[(glucose at 30 min) - (glucose at 0 min)]

^＊^Difference between pre-treatment and post-treatment
