# Supplementary material for: Liraglutide suppresses non-esterified free fatty acids and soluble vascular cell adhesion molecule-1 compared with metformin in patients with recent-onset type 2 diabetes
Source: Cardiovasc Diabetol. 2018 Apr 10;17:53. doi: 10.1186/s12933-018-0701-4 (PMC5891985; doi:10.1186/s12933-018-0701-4)
Supplement: Supplementary file 1 — Additional file 1: Table S1. Changes of plasma lipids and lipoprotein metabolism parameters before and after 8-week treatment with liraglutide and metfotmin. Table S2. Comparisons of body weight, plasma glucose and insulin secretion capacity before and after 8-week treatment between two groups. Table S3. Comparisons of sVCAM-1,sICAM-1,PAI-1 levels before and after 8-week treatment between two groups. Table S4. Spearman rank correlations in ΔNEFA with ΔsVCAM-1, ΔAUCins and ΔI30/ΔG30 after 8-week treatment. [file 12933_2018_701_MOESM1_ESM.zip › Table S3.doc]

**Additional file 1: Table S3 Comparisons of sVCAM-1，sICAM-1，PAI-1 levels before and after 8-week treatment between two groups**

| **Variable** | **Liraglutide Group** | | | **Metformin Group** | | |
| --- | --- | --- | --- | --- | --- | --- |
|  | Pre-  treatment | P Post-  treatment | P -value | Pre-  treatment | Post-  treatment | P- value |
| sVCAM-1  (ng/ml) | 465±136 | 382±131 | 0.013 | 485±122 | 503±182 | 0.708 |
| sICAM-1  (ng/ml) | 168±110 | 172±64 | 0.411 | 113±40 | 103±39 | 0.851 |
| PAI-1  (ng/ml) | 69.52±45.75 | 65.40±40.25 | 0.752 | 51.34±33.87 | 34.36±25.29 | 0.067 |

Data are expressed as mean ±standard

sVCAM-1:soluble vascular cell adhesion molecule-1; sICAM-1:soluble intercellular adhesion molecule-1;PAI-1: plasminogen activator inhibitors-1.
