# Supplementary material for: Liraglutide suppresses non-esterified free fatty acids and soluble vascular cell adhesion molecule-1 compared with metformin in patients with recent-onset type 2 diabetes
Source: Cardiovasc Diabetol. 2018 Apr 10;17:53. doi: 10.1186/s12933-018-0701-4 (PMC5891985; doi:10.1186/s12933-018-0701-4)
Supplement: Supplementary file 1 — Additional file 1: Table S1. Changes of plasma lipids and lipoprotein metabolism parameters before and after 8-week treatment with liraglutide and metfotmin. Table S2. Comparisons of body weight, plasma glucose and insulin secretion capacity before and after 8-week treatment between two groups. Table S3. Comparisons of sVCAM-1,sICAM-1,PAI-1 levels before and after 8-week treatment between two groups. Table S4. Spearman rank correlations in ΔNEFA with ΔsVCAM-1, ΔAUCins and ΔI30/ΔG30 after 8-week treatment. [file 12933_2018_701_MOESM1_ESM.zip › Table S2.doc]

**TabS2 Comparisons of body weight, plasma glucose and insulin secretion capacity before and after 8-week treatment between two groups**

| **Variable** | **Liraglutide Group** | | | **Metformin Group** | | |
| --- | --- | --- | --- | --- | --- | --- |
|  | Pre-  treatment | P Post-  treatment | P -value | Pre-  treatment | P Post-  treatment | P -value |
| BW(Kg) | 81±17 | 78±16 | 0.001 | 72±11 | 70±11 | 0.063 |
| BMI(Kg/m^2^) | 28.63±3.86 | 27.67±3.62 | 0.001 | 26.16±3.10 | 25.62±2.97 | 0.087 |
| WC(cm) | 92±12 | 88±11 | 0.001 | 88±8 | 85±8 | 0.002 |
| FPG  (mmol/L) | 9.40±2.32 | 7.33±2.06 | 0.024 | 8.45±1.57 | 6.67±1.26 | 0.001 |
| 30minPG  (mmol/L) | 15.43±2.96 | 11.46±3.61 | 0.003 | 13.03±2.72 | 12.13±2.07 | 0.078 |
| 60minPG  (mmol/L) | 18.19±3.60 | 14.64±3.86 | 0.012 | 16.43±4.13 | 15.38±3.63 | 0.193 |
| 120minPG  (mmol/L) | 17.68±4.38 | 12.16±5.78 | 0.002 | 14.47±5.05 | 12.43±5.34 | 0.081 |
| Fasting insulin  (mIU/L) | 104  (51,123) | 113  (54,171) | 0.015 | 76  (60, 150) | 87  (41, 175) | 0.650 |
| 30min insulin  (mIU/L) | 271  (136, 298) | 291  (173, 333) | 0.307 | 211  (118, 444) | 217  (158, 387) | 0.865 |
| 60min insulin  (mIU/L) | 326  (165, 441) | 471  (334, 717) | 0.005 | 368  (242, 731) | 396  (277, 918) | 0.932 |
| 120min insulin  (mIU/L) | 401  (193, 560) | 500  (367, 960) | 0.047 | 475  (286, 1060) | 542  (208, 866) | 0.460 |
| AUCins(mIU/L) | 648  (321, 742) | 738  (451, 1118) | 0.005 | 615  (381, 1167) | 594  (357, 1216) | 0.460 |
| ΔI30/ΔG30 | 24.94  (7.78, 38.89) | 31.13  (17.67, 59.09) | 0.031 | 30.18  (10.4,53.75) | 21.96  (14.42,40.93) | 0.650 |
| HbA_1_c(%) | 8.49±1.81 | 6.92±0.81 | 0.002 | 7.77±1.44 | 6.43±0.71 | 0.001 |

Data are expressed as mean ±standard deviation or median( interquartile rang).

BW: body weight; BMI: body mass index; WC:waist circumference; FPG:fasting plasma glucose; AUCins: insulin area under the curve; ΔI30/ΔG30= [(insulin at 30 min) - (insulin at 0 min)]/[(glucose at 30 min) - (glucose at 0 min)]; HbA_1_c:glycated haemoglobin.
