# Supplementary material for: Liraglutide suppresses non-esterified free fatty acids and soluble vascular cell adhesion molecule-1 compared with metformin in patients with recent-onset type 2 diabetes
Source: Cardiovasc Diabetol. 2018 Apr 10;17:53. doi: 10.1186/s12933-018-0701-4 (PMC5891985; doi:10.1186/s12933-018-0701-4)
Supplement: Supplementary file 1 — Additional file 1: Table S1. Changes of plasma lipids and lipoprotein metabolism parameters before and after 8-week treatment with liraglutide and metfotmin. Table S2. Comparisons of body weight, plasma glucose and insulin secretion capacity before and after 8-week treatment between two groups. Table S3. Comparisons of sVCAM-1,sICAM-1,PAI-1 levels before and after 8-week treatment between two groups. Table S4. Spearman rank correlations in ΔNEFA with ΔsVCAM-1, ΔAUCins and ΔI30/ΔG30 after 8-week treatment. [file 12933_2018_701_MOESM1_ESM.zip › TableS1.doc]

**TabS1 Changes of plasma lipids and lipoprotein metabolism parameters before and after 8-week treatment with liraglutide and metformin**

| **Variable** | **Liraglutide Group** | | | **Metformin Group** | | |
| --- | --- | --- | --- | --- | --- | --- |
|  | Pre-  treatment | P Post-  treatment | P -value | Pre-  treatment | P Post-  treatment | P -value |
| TC  (mmol/L) | 4.79±0.87 | 4.64±1.01 | 0.376 | 5.02±0.79 | 4.45±0.28 | 0.006 |
| 120minTC  (mmol/L) | 4.45±1.02 | 4.11±0.88 | 0.139 | 4.55 ±0.81 | 4.10±1.21 | 0.036 |
| ΔTC  (mmol/L) |  |  |  | -0.43  (-0.81,-0.20) | -0.28  (-0.71,-0.03) | 0.394 |
| TG  (mmol/L) | 1.82±0.74 | 1.75±0.91 | 0.652 | 1.99±0.74 | 1.81±0.96 | 0.286 |
| 120minTG  (mmol/L) | 2.07±1.23 | 1.67±0.84 | 0.215 | 1.97±0.68 | 1.79±0.87 | 0.172 |
| LDL-C  ( mmol/L) | 3.61±0.82 | 3.53±0.88 | 0.654 | 3.78±0.68 | 3.18±0.88 | 0.012 |
| 120min LDL-C  (mmol/L) | 3.16±0.70 | 2.88±0.68 | 0.100 | 3.28±0.70 | 2.76±0.91 | 0.008 |
| ΔLDL-C  (mmol/L) |  |  |  | -0.52  (-0.91,-0.13) | -0.42  (-0.73, -0.03) | 0.609 |
| HDL-C  ( mmol/L) | 1.12±0.29 | 1.16±0.26 | 0.250 | 1.20±0.26 | 1.29±0.31 | 0.226 |
| 120min HDL-C  (mmol/L) | 1.04±0.35 | 0.99±0.26 | 0.398 | 1.07±0.23 | 1.18±0.37 | 0.178 |
| NEFA  (μmol/Ll) | 620±468 | 452±177 | 0.181 | 485±225 | 474±125 | 0.855 |
| 120min NEFA  (μmol/L) | 155±125 | 99±73 | 0.026 | 101±53 | 107±59 | 0.650 |

Data are expressed as mean ±standard deviation or median( interquartile rang).

TC: total cholesterol;TG: triglycerides; LDL-C: low-density lipoprotein cholesterol;HDL-C: high-density lipoprotein cholesterol; NEFA:non-esterified fatty acids;

Δ=120min value-fasting value.
